# Mental health disorders among ovarian cancer survivors in a population‐based cohort

**DOI:** 10.1002/cam4.4976

**Published:** 2022-06-30

**Authors:** Siqi Hu, David Baraghoshi, Chun‐Pin Chang, Kerry Rowe, John Snyder, Vikrant Deshmukh, Michael Newman, Alison Fraser, Ken Smith, Anita R. Peoples, David Gaffney, Mia Hashibe

**Affiliations:** ^1^ Huntsman Cancer Institute Salt Lake City Utah USA; ^2^ Division of Public Health, Department of Family and Preventive Medicine University of Utah School of Medicine Salt Lake City Utah USA; ^3^ Intermountain Healthcare Salt Lake City Utah USA; ^4^ University of Utah Health Sciences Center Salt Lake City Utah USA; ^5^ Pedigree and Population Resource, Population Sciences Huntsman Cancer Institute Salt Lake City Utah USA; ^6^ Department of Population Health Sciences University of Utah School of Medicine Salt Lake City Utah USA; ^7^ Department of Radiation Oncology University of Utah School of Medicine Salt Lake City Utah USA

**Keywords:** mental health, mortality, ovarian neoplasms, quality of life, risk factors

## Abstract

**Background:**

Ovarian cancer is the fifth most common female cancer in the United States. There have been very few studies investigating mental health diagnoses among ovarian cancer survivors with long‐term follow up. The aim of this study is to examine the incidence of mental illness among ovarian cancer survivors compared to a general population cohort. A secondary aim is to investigate risk factors for mental illnesses among ovarian cancer survivors.

**Patients and methods:**

Cohorts of 1689 ovarian cancer patients diagnosed between 1996 and 2012 and 7038 women without cancer matched by age and birth state from the general population were identified. Mental health diagnoses were identified from electronic medical records and statewide healthcare facilities data. Cox proportional hazard models were used to estimate hazard ratios (HRs).

**Results:**

Ovarian cancer survivors experienced increased risks of mental illnesses within the first 2 years after cancer diagnosis (HR = 3.55, 95% CI = 3.04–4.14). The risks of depression among ovarian cancer survivors were nearly 3‐fold within the first 2 years of cancer diagnosis (HR = 2.59, 95% CI = 1.94–3.47), and 1.69‐fold at 2–5 years after cancer diagnosis (HR = 1.69, 95% CI = 1.18–2.42). Ovarian cancer survivors experienced an 80% increased risk of death with a mental illness diagnosis (HR = 1.80, 95% CI = 1.48–2.18) and a 94% increased risk of death with a depression diagnosis (HR = 1.94, 95% CI = 1.56–2.40).

**Conclusions:**

Higher risks of mental illnesses were observed among ovarian cancer survivors throughout the follow‐up periods of 0–2 years and 2–5 years after cancer diagnosis. Multidisciplinary care is needed to monitor and treat mental illnesses among ovarian cancer survivors.

## 1 INTRODUCTION

Ovarian cancer is the fifth most common female cancer and the fourth leading cause of cancer‐related deaths among women in the United States.^1^ Depression, anxiety, and post‐traumatic stress disorder (PTSD) have been investigated in previous studies among ovarian cancer survivors. According to a meta‐analysis including 3623 ovarian cancer patients from 24 studies, the prevalence was 12.7% for depression, and 27.1% for anxiety.^2^ However, in these previous studies, the sample sizes were <800 ovarian cancer survivors and the follow‐up periods were up to 2 years.^3^ The prevalence increased by follow‐up time within 6 months after ovarian cancer diagnosis based on six previous studies’ results.^4^, ^5^, ^6^, ^7^, ^8^, ^9^ Previous studies have suggested that worse physical functioning, younger age, higher cancer stage, and not receiving chemotherapy were risk factors for mental illness among ovarian cancer survivors.^10^, ^11^

There are no prior studies that investigated mental health outcomes comprehensively in a large population‐based ovarian cancer cohort. Furthermore, very few studies have investigated the risk factors for mental illness in ovarian cancer patients.^10^, ^11^

The purpose of the present study is to estimate the incidence of mental health disorders among ovarian cancer survivors in comparison with a general population cohort. A secondary aim is to investigate risk factors for mental illnesses among ovarian cancer survivors.

## 2 METHODS

An initial cohort of 2793 ovarian cancer survivors was identified from the statewide SEER Utah Cancer Registry (UCR). Each ovarian cancer survivor was matched with one to five cancer‐free Utah residents from the Utah Population Database (UPDB). The eligibility criteria were: Women diagnosed with an invasive first primary ovarian cancer at 18 years of age or older, between 1996 and 2012 in the state of Utah (SEER ICD‐O‐3 codes: C569). A total of 206 ovarian cancer patients were excluded: 122 because their cancer stage was missing, 24 because an individual from the general population could not be matched, and 60 because their Utah residence did not exceed 1 year. There were 1689 ovarian cancer survivors in the final study sample. 7038 women were matched with each ovarian cancer survivors by birth state, birth year, and follow‐up time from the non‐cancer general population. This study was approved by the University of Utah's Resource for Genetic and Epidemiologic Research (the oversight committee for the UPDB) and the University of Utah Institutional Review Board.

Outcome data used for this study included statewide ambulatory and inpatient data from the Utah Department of Health and electronic medical record data from Intermountain Health Care and the University of Utah Health Sciences Center. Data from the UPDB included records from the UCR, Utah driver's licenses, vital records, and the Utah Department of Health. Outcome data included all available ICD‐9 diagnosis codes and diagnosis dates for mental illnesses. The Clinical Classification Software developed by the Health Cost and Utilization Project was used to categorize ICD‐9 codes into three levels of specificity (Levels 1–3) (Table 3), with Level 1 being the broadest (e.g., mental illness) down to Level 2 (e.g., mood disorders) and Level 3 being the most specific (e.g., depressive disorders). Follow‐up time for incident cases of each outcome was calculated from the ovarian cancer survivor's initial cancer diagnosis to the date of diagnosis, last date of follow‐up, or date of death. The first date of follow‐up of the general population was established as the date of diagnosis of their matched ovarian cancer survivors. Individuals who did not have that outcome were censored at the date of last follow‐up if that date fell within the analysis period. Level 3 outcomes diagnosed prior to the start of each analysis period were considered prevalent cases of those outcomes, and individuals were excluded from the relevant models. Level 2 outcomes were broader and contained multiple disparate conditions; thus, we did not exclude prevalent diagnoses.^12^

Chi‐square tests were used to compare baseline characteristics between the ovarian cancer survivor and general population cohorts. Cox proportional hazards (PH) models were used to calculate hazard ratios (HR) for long‐term mental illness outcomes and risk factors. Cox PH models were adjusted for matching factors (age and birth state), baseline body mass index (bBMI), baseline Charlson Comorbidity Index (bCCI), and race/ethnicity. The bCCI was calculated using all medical record data prior to the date of cancer diagnosis.^13^ Cox proportional hazards models were also used to investigate risk factors for mental illness among ovarian cancer survivors. Kaplan–Meier survival curves were used to survival among patients with and without overall mental illness/depression.

The PH assumption was checked for each model using a test for nonzero slope of the Schoenfeld residuals versus time. Models that were in violation of the PH assumption were then fit with flexible parametric survival models with restricted cubic splines. HRs from the Cox PH models were reported where there were no substantive differences.

Baseline BMI values at least 1 year prior to ovarian cancer diagnosis were calculated from the driver's license records for both cohorts. For individuals missing BMI, values were imputed using fully conditional specification discriminant function methods with cancer diagnosis, bCCI, and age at ovarian cancer diagnosis as covariates. Models were run with and without the imputed values to assure that the inferences did not change due to the imputation of BMI. All statistical tests were two‐sided, and a *p* < 0.05 was considered statistically significant. Analyses were performed using SAS 9.4, except the Flex Spline model using Stata 17.

## 3 RESULTS

Ovarian cancer survivors had a higher proportion of having a baseline comorbidity compared to the general population cohort (36.9% vs. 30.7%) (Table 1). A higher proportion of ovarian cancer survivors were obese at baseline compared to the general population cohort. Based on county level socioeconomic factors, there did not appear to be strong differences between the ovarian cancer survivor and general population cohorts. The mean follow‐up times were 4.75 (SD = 4.94) years for ovarian cancer survivors and 8.76 (SD = 4.83) for the general population cohort. Although matched on birth year, individuals in the general population cohort with a diagnosis of cancer were excluded from this analysis which resulted in differences for age at the end of follow‐up.

**TABLE 1:** Demographic characteristics of ovarian cancer survivor and the general population cohorts

	*N* (%)		*p‐value for Chi‐square*
	Ovarian cancer (*N* = 1689)	General population (*N* = 7038)	
Age at end of follow‐up (years)			
<44	139 (8.2)	466 (6.6)	<0.001
45–54	233 (13.2)	673 (9.6)	
55–64	391 (23.2)	1417 (20.1)	
65–74	409 (24.2)	1687 (24.0)	
75–84	379 (22.4)	1589 (22.6)	
85+	148 (8.8)	1206 (17.1)	
Ethnicity			
Hispanic	121 (7.1)	392 (5.6)	0.5708
Non‐Hispanic	1564 (92.6)	4766 (67.7)	
Race			
White	1656 (98.1)	6591 (93.7)	<0.001
Black	2 (0.1)	22 (0.3)	
Native American	8 (0.5)	40 (0.6)	
Asian	12 (0.7)	120 (1.7)	
Pacific Islander	4 (0.2)	30 (0.4)	
Baseline BMI (kg/m^2^)			
<18.5	44 (2.6)	172 (2.4)	0.147
18.5–24.9	814 (48.2)	3382 (48.1)	
25–29.9	489 (29.0)	2199 (31.2)	
30+	342 (20.3)	1285 (18.3)	
Baseline CCI			
0	1066 (63.11)	4475 (69.3)	<0.001
≥1	623 (36.9)	2163 (30.7)	
Follow‐up period (years)			
0–1	443 (26.2)	68 (1.0)	<0.001
1–5	666 (39.4)	1937 (27.5)	
5–10	314 (18.6)	2313 (32.9)	
10–15	155 (9.2)	1765 (25.1)	
15+	111 (6.6)	955 (13.6)	
Vital status			
Dead	1126 (66.7)	1403 (20.0)	<0.001
Alive	563 (33.3)	5625 (80.1)	
Family history of ovarian cancer^a^			
Yes	168 (11.0)	550 (7.9)	0.206
No	1503 (89.0)	6488 (92.1)	
% Bachelors degree^b^			
<15%	99 (5.9)	441 (6.3)	0.264
15%–24.9%	382 (22.6)	1706 (24.2)	
≥25%	1208 (71.5)	4891 (69.5)	
% Families below poverty^b^			
<7%	1003 (59.4)	4138 (58.8)	0.614
7%–9%	492 (29.1)	2030 (28.8)	
>9%	194 (11.5)	870 (12.4)	
Median family income^b^			
<50,000	285 (16.9)	1224 (17.4)	0.614
≥50,000	1404 (83.1)	5814 (82.6)	

Abbreviations: BMI, body mass index; CCI, Charlson comorbidity index.

^a^ Included family history of first, second and third degree relatives.

^b^ At county level.

Approximately 67.6% of the ovarian cancer survivors were diagnosed at distant stage, and 51.7% with a histology subtype of high‐grade serous (Table 2). The majority of ovarian cancer survivors (47.5%) received combined treatment of surgery and chemotherapy.

**TABLE 2:** Clinical characteristics of ovarian cancer survivors

	Ovarian cancer (*N* = 1689) (*n* [%])
Diagnosis year	
1996–1999	369 (21.9)
2000–2004	401 (23.7)
2005–2009	382 (22.6)
2010–2012	537 (31.8)
Age at diagnosis	
<40	144 (8.53)
40–49	240 (14.2)
50–59	377 (22.3)
60–69	365 (21.6)
70–79	363 (21.5)
80+	200 (11.8)
Cancer stage	
Local	295 (17.5)
Regional	253 (15.0)
Distant	1141 (67.6)
Histology	
High‐grade serous	873 (51.7)
Low‐grade serous	17 (1.0)
Endometrioid	143 (8.5)
Mucinous	115 (6.8)
Clear cell	94 (5.6)
Carcinosarcoma	38 (2.3)
Carcinoma, NOS	245 (14.5)
Other^a^	164 (9.7)
Treatment	
Surgery only	448 (26.5)
Radiation only	60 (3.6)
Chemotherapy only	155 (9.2)
Surgery and chemotherapy	803 (47.5)
Other^b^	16 (1.0)
No treatment	207 (12.3)

^a^ Including Malignant Brenner, Mixed, Non‐specific.

^b^ Including combination of radiation and surgery, combination of radiation and chemotherapy.

**FIGURE 1:**
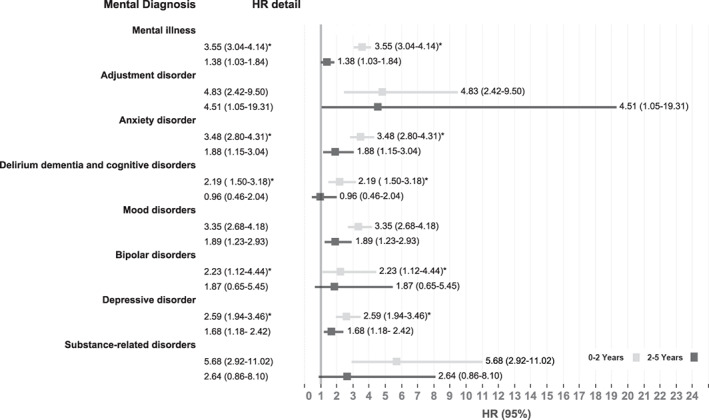
Adjusted hazard ratios for mental illness among ovarian cancer survivors in comparison to the general population cohort. HR, hazard ratio.

An overview of adjusted HRs is shown in Figure 1. The risk for any mental health diagnosis of ovarian cancer survivors was increased at 0–2 years after cancer diagnosis in comparison with the general population cohort (HR = 3.55, 95% CI = 3.04–4.14; Table 3). The proportion of ovarian cancer patients diagnosed with any mental health diagnosis was 34.2% at 0–2 years, 27.8% at 2–5 years, and 36.3% at >5 years from diagnosis. Ovarian cancer survivors had increased risks of depression, anxiety, and adjustment disorders in comparison with the general population cohort. The risks of depression among ovarian cancer survivors were 3‐fold at 0–2 years (HR = 2.59, 95% CI = 1.94–3.47), and 1.69‐fold at 2–5 years after cancer diagnosis (HR = 1.69, 95% CI = 1.18–2.42). The risk of anxiety disorder among ovarian cancer survivors was 3.54‐fold at 0–2 years (HR = 3.48, 95% CI = 2.81–4.31), and 1.86‐fold at 2–5 years (HR = 1.88, 95% CI = 1.15–3.05). Table S1 shows similarly increased risks for mental health disorders without the adjustment of matching factors (age and birth state).

**TABLE 3:** Hazard ratios for mental illness among ovarian cancer survivors in comparison to the general population cohort

Diagnosis (CCS code)^d^	0–2 years after cancer diagnosis			2–5 years after cancer diagnosis			5+ years after cancer diagnosis		
	Cancer cases (*n*[%])	Gen. (*n*[%])	Adj.HR (95% CI)	Cancer cases (*n*[%])	Gen. (*n*[%])	Adj.HR (95% CI)	Cancer cases (*n*[%])	Gen. (*n*[%])	Adj.HR (95% CI)
Mental illness (5)^b^^,^^c^	578 (34.2)	1143 (16.2)	**3.55^a^(3.04–4.14)**	285 (27.8)	827 (19.6)	**1.38 (1.03–1.84)**	168 (36.3)	611 (33.7)	0.86 (0.59–1.24)
Adjustment disorder (5.1)	19 (1.1)	14 (0.3)	**4.83^a^(2.46–9.50)**	12 (1.2)	15 (0.4)	**4.52 (1.06–19.31)**	7 (1.5)	14 (0.8)	2.35 (0.38–14.74)
Anxiety disorder (5.2)	234 (13.9)	402 (5.7)	**3.48^a^(2.81–4.31)**	119 (11.6)	317 (7.5)	**1.88 (1.15–3.05)**	83 (17.9)	299 (16.5)	1.02 (0.61–1.68)
Delirium dementia and cognitive disorders (5.4)	65 (3.9)	206 (2.9)	**2.19^a^(1.50–3.18)**	29 (2.8)	94 (2.2)	0.96 (0.46–2.00)	27 (5.8)	121 (6.7)	0.87 (0.44–1.73)
Mood disorders (5.8)	275 (16.3)	628 (8.9)	**3.35^a^(2.68–4.18)**	160 (15.6)	473 (11.2)	**1.90 (1.23–2.93)**	101 (21.8)	355 (19.6)	0.94 (0.55–1.60)
Bipolar disorders (5.8.1)	22 (1.3)	57 (0.8)	**2.23^a^(1.12–4.44)**	15 (1.5)	38 (0.9)	1.87 (0.64–5.45)	9 (1.9)	39 (2.2)	1.59 (0.30–8.33)
Depressive disorder (5.8.2)	264 (15.6)	602 (8.6)	**2.59 (1.94**–**3.47)**	156 (15.2)	463 (11.0)	**1.69 (1.18–2.42)**	100 (21.6)	345 (19.0)	1.14 (0.67–1.94)
Schizophrenia and other psychotic disorders (5.10)	36 (2.1)	67 (1.0)	**4.11 (1.81**–**9.32)**	22 (2.2)	36 (0.9)	**3.65 (1.45–9.20)**	20 (4.3)	55 (3.0)	1.09 (0.44–2.71)
Alcohol‐related disorders (5.11)	17 (1.0)	25 (0.4)	2.93 (0.87–9.83)	4 (0.4)	33 (0.8)	0.40 (0.05–3.10)	5 (1.1)	27 (1.5)	0.76 (0.13–4.30)
Substance‐related disorders (5.12)	37 (2.2)	59 (0.8)	**5.68^a^(2.92–11.02)**	16 (1.6)	32 (0.8)	2.64 (0.86–8.10)	8 (1.7)	38 (2.1)	0.88 (0.23–3.40)
Suicide and intentional self‐inflicted injury (5.13)	2 (0.1)	14 (0.2)	0.76 (0.09–6.51)	3 (0.3)	17 (0.5)	0.63 (0.09–4.48)	1 (0.2)	17 (0.9)	NA

*Note*: Bold value indicates statistical significance (*p* < 0.05).

All hazard ratios adjusted for age at diagnosis, birth state, baseline BMI, baseline CCI, and ethnicity/race. CI, confidence interval.

^a^ Proportional hazard assumption not met; flexible spline model used.

^b^ Some outcomes that developed or diagnosed before adulthood were excluded from this table: Attention deficit conduct and disruptive behavior disorder (5.3), Developmental disorder (5.5) and its related disorders, Disorders usually diagnosed in infancy childhood or adolescence (5.6) and its related disorders,

^c^ Some outcomes that had limited observations were excluded from this table: Personality disorder (5.9), Screening and history of mental health and substance abuse codes (5.14), Miscellaneous mental disorder (5.15) and its related disorders.

^d^ The CSS codes were used to categorize ICD‐9 codes into three levels of specificity (Levels 1–3), with level 1 being the broadest (e.g., mental illness(5)) down to level 2 (e.g., mood disorders (5.8)) and level 3 being the most specific (e.g., depressive disorders (5.8.2)).”

Elevated risk for adjustment disorders was observed among ovarian cancer survivors compared with the general population cohort between 0–2 years ((HR = 4.83, 95% CI = 2.46–9.50) and between 2–5 years (HR = 4.52, 95% CI = 1.06–19.31). Increased risk for bipolar disorder diagnosis was observed among ovarian cancer at 0–2 years (HR = 2.23, 95% CI = 1.12–4.44) compared to the general population cohort. Although not statistically significant, an increased risk of bipolar disorder was also observed at 2–5 years (HR = 1.87, 95% CI = 0.64–5.45). Ovarian cancer survivors were more likely to be diagnosed with schizophrenia and other psychotic disorders during 0–2 years (HR = 4.11, 95% CI = 1.81–9.32) and 2–5 years (HR = 3.65, 95% CI = 1.45–9.20) after cancer diagnosis. The risk of substance‐related disorder among ovarian cancer survivors were also increased at 0–2 years (HR = 5.68, 95% CI = 2.92–11.02) and 2–5 years after cancer diagnosis (HR = 2.64, 95% CI = 0.86–8.10).

Cancer treatment and later diagnosis year were associated with an increased risk of any mental illness at 0–2 years after cancer diagnosis among ovarian cancer survivors (Table 4). Distant‐stage cancer was an important risk factor compared to early‐stage for both mental illness and depression among ovarian cancer survivors in all time periods. Ovarian cancer patients who had a mucinous histology subtype had 47% decreased risk of any mental illness and 67% decreased risk of depression at 0–2 years, compared to those with high‐grade serous histology subtype. In addition, a bCCI score of 1+ was associated with the increased risk of depression at 0–2 years after cancer diagnosis. Older age at cancer diagnosis (>60 years old) was associated with the risk of any mental illness and depression >5 years after cancer diagnosis compared to younger age at diagnosis (=<60 years old). We also investigated the contribution of race, ethnicity, rural residence, and socioeconomic factors to mental illness risks, but did not observe any associations (data not shown).

**TABLE 4:** Risk factors for mental illness and depression among ovarian cancer survivors

Factors	0–2 years after cancer diagnosis				2–5 years after cancer diagnosis				>5 years after cancer diagnosis			
	Mental illness		Depression		Mental illness		Depression		Mental illness		Depression	
	HR	95% CI	HR	95% CI	HR	95% CI	HR	95% CI	HR	95% CI	HR	95% CI
Treatment^a^												
Surgery	Reference		Reference		Reference		Reference		Reference		Reference	
Chemo	1.29	(0.81–2.05)	0.88	(0.45–1.72)	0.96	(0.38–2.43)	1.97	(0.78–4.98)				
Surgery + Chemo	**1.39**	**(1.01–1.90)**	1.30	(0.85–1.97)	1.33	(0.81–2.16)	1.66	(0.92–3.00)	1.20	(0.63–2.29)	1.02	(0.50–2.06)
No treatment	0.84	(0.20–3.55)			4.82	(0.57–40.47)	8.09	(0.97–67.84)				
Cancer stage (SEER)^b^												
Local	Reference		Reference		Reference		Reference		Reference		Reference	
Regional	1.50	(0.96–2.34)	0.75	(0.41–1.38)	1.17	(0.61–2.22)	1.62	(0.81–3.25	1.29	(0.54–3.13)	1.72	(0.69–4.29)
Distant	**2.06**	**(1.44–2.95)**	**1.59**	**(1.03–2.45)**	**1.68**	**(1.03–2.76)**	1.70	(0.94–3.08)	1.82	(0.98–3.41)	**2.81**	**(1.33– 5.91)**
Diagnosis year^c^												
1996–1999	Reference		Reference		Reference		Reference		Reference		Reference	
2000–2003	1.27	(0.90–1.79)	0.79	(0.45–1.39)	1.32	(0.75–2.32)	1.18	(0.60–2.32)	0.79	(0.41–1.54)	1.03	(0.49–2.17)
2003–2007	1.26	(0.88–1.82)	1.62	(0.98–2.68)	1.35	(0.75–2.41)	0.82	(0.38–1.74)	0.46	(0.18–1.15)	0.58	(0.22–1.53)
2008–2012	**1.49**	**(1.06–2.09)**	1.54	(0.94–2.51)	1.15	(0.65–2.05)	1.53	(0.81–2.89)			0.29	(0.04–2.31)
Histology^d^												
High‐grade serous	Reference		Reference		Reference		Reference		Reference		Reference	
Low‐grade serous	0.65	(0.21–2.07)			0.75	(0.18–3.14)	1.85	(0.55–6.22)	**4.95**	**(1.50–16.36)**	1.58	(0.30–8.34)
Endometrioid	0.72	(0.46–1.12)	0.564	(0.31–1.04)	0.49	(0.23–1.03)	0.40	(0.16–1.01)	1.05	(0.46–2.39)	0.67	(0.28–1.61)
Mucinous	**0.53**	**(0.30–0.95)**	**0.33**	**(0.13–0.82)**	0.82	(0.38–1.76)	1.09	(0.50–2.41)	1.11	(0.48–2.54)	0.80	(0.29–2.16)
Clear cell	0.75	(0.44–1.28)	0.55	(0.26–1.19)	0.94	(0.42–2.09)	1.20	(0.50–2.88)	0.28	(0.03–2.24)	0.30	(0.04–2.29)
Carcinosarcoma	1.40	(0.65–3.00)	1.11	(0.41–3.05)	0.79	(0.11–5.81)						
Carcinoma, NOS	1.34	(0.96–1.86)	1.06	(0.65–1.73)	0.68	(0.29–1.61)	0.80	(0.28–2.26)	1.64	(0.51–5.22)	0.23	(0.03–1.74)
Other^h^	**0.60**	**(0.37–0.97)**	0.57	(0.30–1.06)	1.00	(0.51–1.95)	0.98	(0.44–2.18)	0.62	(0.17–2.27)	0.62	(0.20–1.95)
Baseline CCI^e^												
0	Reference		Reference		Reference		Reference		Reference		Reference	
1+	1.28	(0.99–1.66)	**1.54**	**(1.10–2.14)**	1.51	(0.98–2.33)	1.38	(0.83–2.27)	1.22	(0.59–2.53)	1.32	(0.62–2.80)
Baseline BMI (kg/m^2^)^f^												
<18.5	1.09	(0.53–2.23)	1.30	(0.53–3.24)	0.87	(0.21–3.65)	2.13	(0.64–7.05)	1.02	(0.14–7.68)	2.69	(0.61–11.85)
18.5–24.9	Reference		Reference		Reference		Reference		Reference		Reference	
25–29.9	1.03	(0.79–1.35)	1.191	(0.83–1.72)	1.12	(0.70–1.78)	1.32	(0.76–2.29)	0.67	(0.34–1.32)	0.97	(0.48–1.94)
>30	0.91	(0.67–1.24)	1.15	(0.77–1.74)	1.11	(0.66–1.85)	1.28	(0.71–2.33)	1.21	(0.60–2.43)	0.54	(0.20–1.45)
Age at diagnosis (y)^g^												
<60	Reference		Reference		Reference		Reference		Reference		Reference	
≥ 60	0.89	(0.71–1.13)	0.75	(0.54–1.02)	1.25	(0.83–1.88)	1.16	(0.73–1.85)	**2.31**	**(1.31– 4.08)**	**1.89**	**(1.01– 3.54)**

*Note*: Bold value indicates statistical significance (*p* < 0.05).

Abbreviations: BMI, body mass index; CCI, Charlson Comorbidity Index; CI, confidence interval; HR, hazard ratio.

^a^ Adjusted for race/ethnicity, baseline BMI, baseline CCI, cancer stage, age at diagnosis, income and education.

^b^ Adjusted for race/ethnicity, baseline BMI, diagnosis year, age at diagnosis, income and education.

^c^ Adjusted for baseline BMI and baseline CCI.

^d^ Adjusted for race/ethnicity, baseline BMI, age at diagnosis, diagnosis year, income and education.

^e^ Adjusted for race/ethnicity and age at diagnosis.

^f^ Adjusted for race/ethnicity, diagnosis age, income and education.

^g^ Adjusted for race/ethnicity and cancer stage.

^h^ Including Malignant Brenner, Mixed, Non‐specific.

**FIGURE 2:**
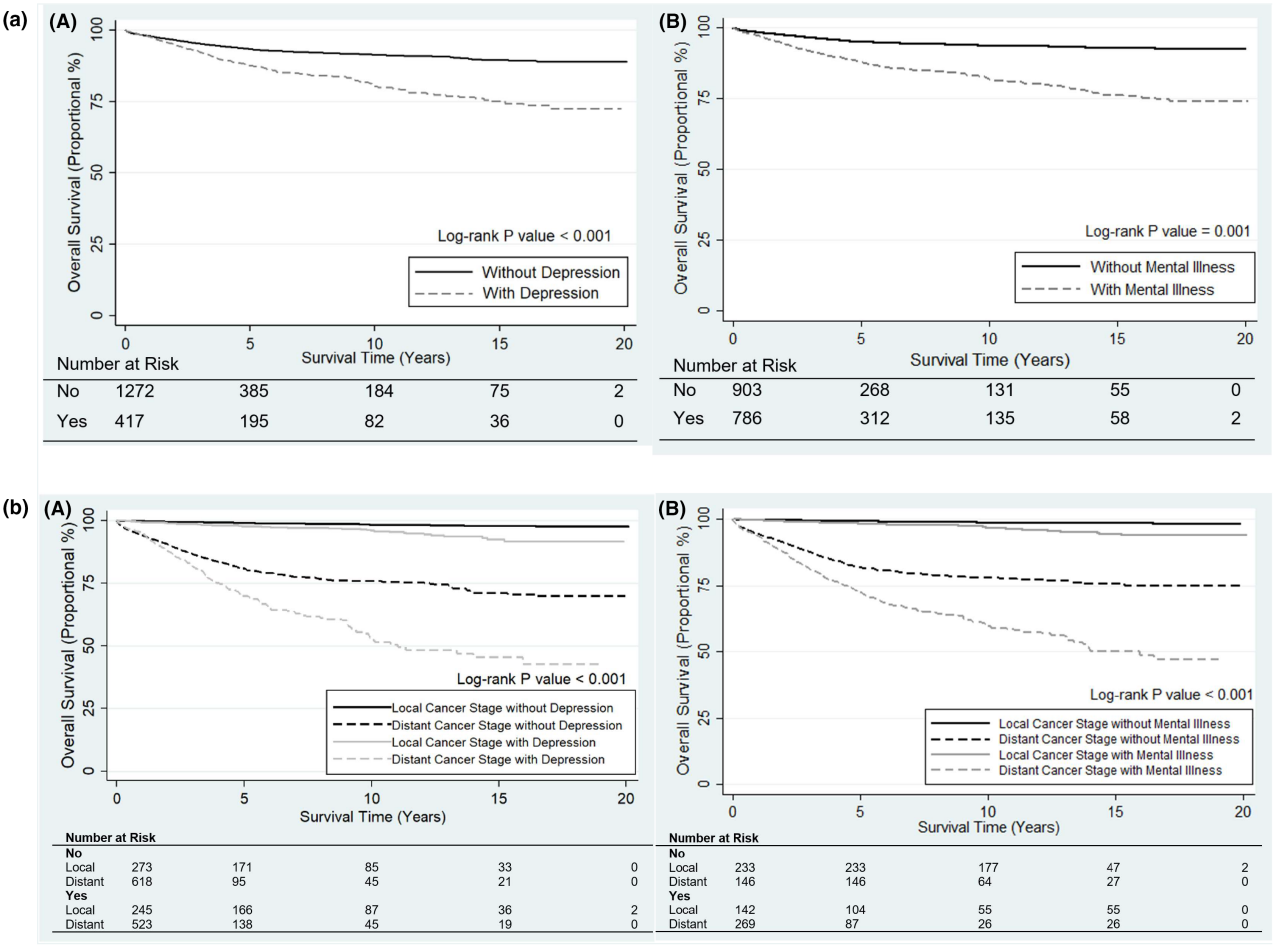
(a) Kaplan–Meier survival curves for ovarian cancer survivors with/without depression (A) and Any mental illness (B) adjusting for baseline BMI, Baseline CCI and age at Diagnosis. (b) Kaplan–Meier survival curves for ovarian cancer survivors with/without depression alone (A) and Any mental illness (B), Stratified by cancer stage, adjusting for baseline BMI, Baseline CCI and age at diagnosis. BMI, body mass index; CCI, Charlson comorbidity index.

Figure 2(a) shows the overall survival among ovarian cancer patients who were diagnosed at any point during follow‐up with any mental illness or depression and patients who were not diagnosed with these diseases. After adjusting for age at diagnosis, bBMI and bCCI, ovarian cancer survivors with any mental illness had an increased risk of death (HR = 1.80, 95% CI = 1.48–2.18) and ovarian cancer survivors with depression also had increased risk of death (HR = 1.94, 95% CI = 1.56–2.40) compared to ovarian cancer patients without mental illnesses or depression. By stratifying by the cancer stage, Figure 2(b) indicates the significant disparity of the overall survival between ovarian cancer patients with or without any mental illness/depression during follow‐up period among different cancer stages.

## 4 DISCUSSION

To our knowledge, this is the first study estimating the risk of mental illness diagnoses among ovarian cancer survivors compared to a general population of women without cancer, and the first to follow ovarian cancer patients over both short‐term and long‐term periods. Our analyses show that ovarian cancer survivors had increased risks of various mental illnesses compared to the general population, as well as higher risks of psychological disorders including adjustment disorder, anxiety disorder, delirium dementia, and cognitive disorder, mood disorder (bipolar and depression disorder), schizophrenia and other psychotic disorders, and substance‐related disorders. We also observed that distant‐stage disease was a significant risk factor for both mental illnesses overall and depression among ovarian cancer patients. Ovarian cancer survivors with mental illnesses or depression had lower survival time than those without these diagnoses. Our findings provide further evidence of the psychological burden among ovarian cancer survivors and highlight the need to improve mental health support for ovarian cancer patients.

We identified significantly increased risks of mental illnesses among ovarian cancer survivors compared to the general population, especially for depression and anxiety disorders. Similar observations regarding the high incidence of depression and anxiety within 2 years after cancer diagnosis were reported in previous studies.^14^, ^15^ Similar to results from our study, a prior study suggested that ovarian cancer patients experienced lower risks of depression and anxiety after a long time (>30 months) since cancer diagnosis.^14^, ^15^, ^16^ Some potential reasons behind lower risks of mental illnesses after a long time since cancer diagnosis: High mortality of ovarian cancer survivors with mental illnesses, or possibly adjustment of emotion and greater acceptance of their ovarian cancer diagnosis.^14^, ^15^, ^16^ Previous studies indicated that psychological distress may be a powerful indicator to predict mortality of cancer in general.^17^, ^18^ Our findings provide further evidence that ovarian cancer survivors with depression had a 94% increase in the risk of death, which indicates that mental illness diagnoses may be an important prognostic factor for ovarian cancer patients.

Our findings are consistent with prior studies indicating that cancer patients experience elevated risks of adjustment disorder.^19^, ^20^ In our study, the risk of adjustment disorder in cancer group was nearly five‐times higher than the general population cohort at 0–2 years, and the relative risk was higher at 2–5 years after cancer diagnosis. However, the essential features of adjustment disorder may be resolved if the individual is able to adapt to the situation and rarely lasts longer than six months,^31^ which is in contrast to the long‐term effect of adjustment disorder observed in our study. The possible reason may be that ovarian cancer patients continue to experience various events after cancer diagnosis, including medication consultation, massive surgery, and severe side effects of chemotherapy and radiation therapy.^21^

Long‐term substance abuse may be a trigger for bipolar disorder and schizophrenia.^22^ Our study results suggested that ovarian cancer survivors were three‐time more likely to experience bipolar disorder and were four‐times more likely to develop schizophrenia at 0–5 years compared to the general population. Schizophrenia and bipolar disorders are commonly diagnosed during adolescence and early adulthood.^23^, ^24^ Prior studies also reported that more than 69% of schizophrenia and bipolar disorders were misdiagnosed or not receiving appropriate care.^25^, ^26^ Thus, ovarian cancer survivors may have had more health‐care visits where perhaps they were diagnosed with pre‐existing psychiatric disorders,^27^ which may explain the increased risks of schizophrenia and bipolar disorders in our study. Thus, due to the high potential of medication side effects and increased suicide rates of schizophrenia and bipolar disorder,^28^ early identification of patients in need of psychological treatment is important for both healthcare professionals and ovarian cancer patients.

Among risk factors for mental illnesses, ovarian cancer survivors who receive combined treatment of surgery and chemotherapy had an elevated risk of any mental illness. This finding is consistent with the existing studies that ovarian cancer patients with surgery and adjuvant chemotherapy were more likely to experience mental disorders due to poor body image, side effects from treatment such as nausea and fatigue, and other fatal diseases.^14^ In addition, the elevated risks of mental illnesses in ovarian cancer survivorship may be explained by the long‐term side effect of chemotherapy, such as pain, dyspnea, appetite loss, and neuropathy symptoms, lower quality of life, which may last for up to 12 years.^29^ Previous studies also reported ovarian cancer patients with surgical oophorectomy were significantly more likely to report mental problems,^30^ although these differences were not statistically significant in our study.

Compared to local‐stage ovarian cancer, our study suggests that distant‐stage ovarian cancer may be a potential risk factor of any mental illness and depression. Our results are consistent with findings from prior research reporting that ovarian cancer patients with more advanced or recurrent disease have greater psychological distress,^14^, ^15^ which might be explained by poor treatment response, severe treatment side effects and worse prognosis.^31^, ^32^ In contrast, one previous study reported that the stage of ovarian cancer has no association with worse mental health outcomes; however, the sample size was small (n = 100).^15^ In addition, the reduced risk with mucinous histology subtype was associated with any mental illness and depression, compared with high‐grade serous histology subtype. This was consistent with previous clinical knowledge that early‐stage mucinous ovarian cancer patients have up to 90% survival rate, and surgery alone is often sufficient.^33^ The majority of mucinous ovarian cancer (66.09%) in our study population were diagnosed with early‐stage disease.

We observed that ovarian cancer survivors who have developed mental illness or depression also had increased risks of death. Notably, the mortality risks in our study were higher than the mortality risks in a population‐based study of other types' cancer, which the risks ranged from 1.03 to 1.29, compared to the cancer patients without psychological distress.^34^, ^35^ The reason may be that more than 75% of ovarian cancer patients are diagnosed with advanced cancer stage compared to other cancer patients,^36^ which may cause tremendous mental burden.

The major strength of this study is the population‐based design with a large sample size of over 1600 ovarian cancer survivors. This large population allowed us to study both common and rare mental health outcomes over a long‐term (up to 20 years) follow‐up period. The electronic medical record data from two of the largest health‐care providers in Utah (Intermountain Healthcare and University of Utah Healthcare) improve our capability to capture the important information that included patients at physical and psychological functioning. Additionally, this study does not rely on self‐reported data, which minimizes recall error and survival bias.

This study also has a number of limitations. First, electronic medical record data led to a risk of coding errors which may overlook the true impact of mental illnesses among ovarian cancer survivors. Second, another limitation is missing bBMI data in some participants, which we calculated by imputation. However, we conducted sensitivity analysis to assure that the imputed BMI value did not influence the inferences in our study. Third, the treatment category on medical records was broad which also limited our analysis. Fourth, the high mortality rate of ovarian cancer patients and long‐term property in our study may still result in survival bias, which could underestimate the impacts of mental illnesses among ovarian cancer survivors. Fifth, recurrence information is not available in this study, which could be a strong risk factor for both mental illness and mortality.

In summary, compared to the general population, the risks of a range of mental disorders were elevated among ovarian cancer survivors. These risks were decreased over the follow up years, but they remained high even in long‐term follow‐up, compared with the general population. We identified several potential determinants of mental illness and depression among ovarian cancer survivors, such as the treatment regimen including surgery and chemotherapy, distant cancer stage, mucinous histology subtype, and higher baseline CCI. This finding emphasizes the importance of providing psychological support after cancer diagnosis and provides new insight to potentially prolong the lives of ovarian cancer survivors.

## AUTHOR CONTRIBUTIONS

Siqi Hu: Conceptualization and design, formal data analysis, original draft. David Baraghoshi: Data analysis, review and editing. Chun‐Pin Chang, Kerry Rowe, John Snyder, Vikrant Deshmukh, Michael Newman, Alison Fraser, and Ken Smith: Data acquisition and method development. Anita R. Peoples and David Gaffney: Critical revision of the manuscript. Mia Hashibe: Funding acquisition, conceptualization, overall supervision.

## CONFLICT OF INTEREST

The authors declare no potential conflicts of interest.

## FUNDING INFOMATION

This work was supported by grants from the NIH (R21 CA185811, R03 CA159357, M.Hashibe, PI), the Huntsman Cancer Institute, and the Cancer Control and Population Sciences Program (HCI Cancer Center Support Grant P30CA042014). This research was supported by the Utah Cancer Registry, which is funded by the National Cancer Institute‘s SEER Program, Contract No. HHSN261201800016I, the US Center for Disease Control and Prevention's National Program of Cancer Registries, Cooperative Agreement No. NU58DP0063200‐01, with additional support from the University of Utah, and Huntsman Cancer Foundation. Partial support for all datasets within the Utah Population Database is provided by the University of Utah, Huntsman Cancer Institute and the Huntsman Cancer Institute Cancer Center Support grant, P30 CA42014 from the National Cancer Institute.

## Supporting information


Supplementary Table 1

## Data Availability

The data that support the findings of this study are available on request from the corresponding author. The data are not publicly available due to privacy or ethical restrictions.
