# Supplementary material for: Mental health disorders among ovarian cancer survivors in a population‐based cohort
Source: Cancer Med. 2022 Jun 30;12(2):1801–12. doi: 10.1002/cam4.4976 (PMC9883396; doi:10.1002/cam4.4976)
Supplement: Supplementary file 1 — Supplementary Table 1 [file CAM4-12-1801-s001.docx]

**Supplement Table 1. Hazard Ratios for Mental Illness among Ovarian Cancer Survivors in Comparison to the General Population Cohort (Overall Follow-up Period)**

| Diagnosis(CCS Code) | | | Overall Follow-up After Cancer Diagnosis | | | | | | | | |
| --- | --- | --- | --- | --- | --- | --- | --- | --- | --- | --- | --- |
|  |  |  | Cancer Cases(n[%]) | | Gen.(n[%]) | | Adj.HR(95% CI) | | | | |
|  |  |  |  |  |  |  |  |  |  |  |  |
| Mental illness(5) | | | 786 | (46.5) | 2918 | 41.46 | **1.21** |  | **(1.00** | **-** | **1.46)** |
|  | Adjustment disorder(5.1) | | 40 | (2.4) | 78 | (1.1) | **2.99** |  | **(1.38** | **-** | **6.46)** |
|  | Anxiety disorder(5.2) | | 371 | (22.0) | 1318 | (18.7) | **1.42** | ***** | **(1.20** | **-** | **1.69)** |
|  | Delirium dementia and cognitive disorders(5.4) | | 117 | (6.9) | 800 | (11.4) | 0.90 |  | (0.58 | **-** | 1.40) |
|  | Mood disorders(5.8) | | 428 | (25.3) | 1659 | (23.6) | **1.29** | ***** | **(1.09** | **-** | **1.53)** |
|  |  | Bipolar disorders (5.8.1) | 42 | (2.5) | 166 | (2.4) | 1.65 |  | (0.86 | **-** | 3.19) |
|  |  | Depressive disorder (5.8.2) | 417 | (24.7) | 1625 | (23.1) | **2.18** |  | **(1.72** | **-** | **2.77)** |
|  | Schizophrenia and other psychotic disorders (5.10) | | 80 | (4.7) | 308 | (4.4) | **1.80** | ***** | **(1.33** | **-** | **2.44)** |
|  | Alcohol-related disorders (5.11) | | 25 | (1.5) | 119 | (1.7) | 0.73 |  | (0.28 | **-** | 1.91) |
|  | Substance-related disorders (5.12) | | 201 | (3.4) | 259 | (2.9) | 1.27 |  | (0.67 | **-** | 2.41) |
|  | Suicide and intentional self-inflicted injury (5.13) | | 81 | (0.3) | 86 | (1.2) | 0.15 |  | (0.02 | **-** | 0.94) |
| Bold value indicates statistical significance (P<0.05). All hazard ratios adjusted for age at diagnosis, birth state, baseline BMI, baseline CCI, and ethnicity/race. CI, confidence interval. a. Proportional hazard assumption not met; flexible spline model used. b. Some outcomes that developed or diagnosed before adulthood were excluded from this table: Attention deficit conduct and disruptive behavior disorder (5.3), Developmental disorder (5.5) and its related disorders, Disorders usually diagnosed in infancy childhood or adolescence (5.6) and its related disorders,  c. Some outcomes that had limited observations were excluded from this table: Personality disorder (5.9), Screening and history of mental health and substance abuse codes (5.14), Miscellaneous mental disorder (5.15) and its related disorders. | | | | | | | | | | | |
|  |  |  |  |  |  |  |  |  |  |  |  |
|  |  |  |  |  |  |  |  |  |  |  |  |
|  |  |  |  |  |  |  |  |  |  |  |  |
|  |  |  |  |  |  |  |  |  |  |  |  |
|  |  |  |  |  |  |  |  |  |  |  |  |
